# Selective Hydrolysis of Ovalbumin Promoted by Hf(IV)-Substituted Wells-Dawson-Type Polyoxometalate

**DOI:** 10.3389/fchem.2018.00614

**Published:** 2018-12-13

**Authors:** Alexander V. Anyushin, Annelies Sap, Thomas Quanten, Paul Proost, Tatjana N. Parac-Vogt

**Affiliations:** ^1^Laboratory of Bio-Inorganic Chemistry, Department of Chemistry, KU Leuven, Leuven, Belgium; ^2^Laboratory of Molecular Immunology, Department of Microbiology and Immunology, KU Leuven, Leuven, Belgium

**Keywords:** polyoxometalates, ovalbumin, hafnium, Wells-Dawson, protein, hydrolysis

## Abstract

The reactivity and selectivity of Wells-Dawson type polyoxometalate (POM), K_16_[Hf(α_2_-P_2_W_17_O_61_)_2_]·19H_2_O (Hf1-WD2), have been examined with respect to the hydrolysis of ovalbumin (OVA), a storage protein consisting of 385 amino acids. The exact cleavage sites have been determined by Edman degradation experiments, which indicated that Hf1-WD2 POM selectively cleaved OVA at eight peptide bonds: Phe13-Asp14, Arg85-Asp86, Asn95-Asp96, Ala139-Asp140, Ser148-Trp149, Ala361-Asp362, Asp362-His363, and Pro364-Phe365. A combination of spectroscopic methods including ^31^P NMR, Circular Dichroism (CD), and Tryptophan (Trp) fluorescence spectroscopy were employed to gain better understanding of the observed selective cleavage and the underlying hydrolytic mechanism. ^31^P NMR spectra have shown that signals corresponding to Hf1-WD2 gradually broaden upon addition of OVA and completely disappear when the POM-protein molar ratio becomes 1:1, indicating formation of a large POM/protein complex. CD demonstrated that interactions of Hf1-WD2 with OVA in the solution do not result in protein unfolding or denaturation even upon adding an excess of POM. Trp fluorescence spectroscopy measurements revealed that the interaction of Hf1-WD2 with OVA (*K*_*q*_ = 1.1 × 10^5^ M^−1^) is both quantitatively and qualitatively slightly weaker than the interaction of isostructural Zr-containing Wells-Dawson POM (Zr1-WD2) with human serum albumin (HAS) (*K*_*q*_ = 5.1 × 10^5^ M^−1^).

## Introduction

**Figure 1:**
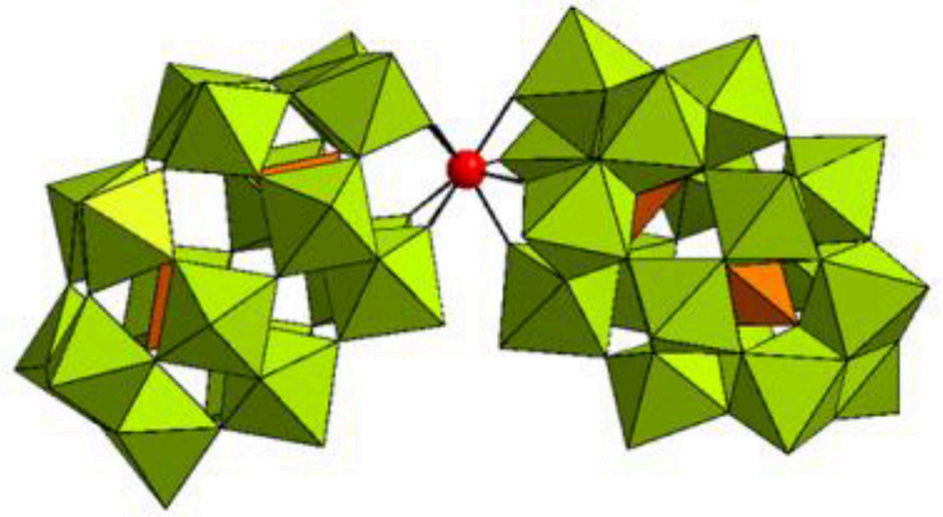
Combined polyhedral/ball-and-stick representation of [Hf(α_2_-P_2_W_17_O_61_)_2_]^16−^ in K_16_[Hf(α_2_-P_2_W_17_O_61_)_2_]·19H_2_O. WO_6_ octahedrons are shown in olive green, PO_4_ tetrahedrons are shown in orange, and the Hf(IV) center is represented as a red sphere.

Polyoxometalates (POMs) are a diverse class of metal-oxygen clusters with a wide range of tunable parameters such as their size, polarity, charge density, solubility and acid-strength (Pope, 1983; Long et al., 2010). Their easily modifiable properties (Sullivan et al., 2018) make them highly applicable in various research domains such as modern catalysis (Lv et al., 2012; Wang and Yang, 2015; Huang et al., 2018; Martin-Sabi et al., 2018; Yu et al., 2018), green energy production and storage (Chen et al., 2017, 2018), material science (Bijelic and Rompel, 2015; Sun et al., 2015; Boyd et al., 2017; Vilà-Nadal and Cronin, 2017; Zhan et al., 2017; Luo et al., 2018), bio-mimics design (Kulikov et al., 2017), and medicine (Bijelic et al., 2018a,b). Currently nearly 80% of all patents concerning POMs are related to catalysis, reflecting their importance as catalysts in various applications (Wang and Yang, 2015; Weinstock et al., 2018). The interest in developing POMs as catalysts is to a large extent related to their rich structural chemistry which allows fine-tuning of their reactivity and other chemical properties such as redox potentials and acidity. The majority of POM-based catalysis has been focused on Brønsted catalyzed reactions and oxidations (Zhou et al., 2014). However, the use of POMs as catalysts in Lewis acid catalyzed reactions has recently also gained in importance. Lewis acid active POMs are typically obtained by incorporating highly charged metal cations into the lacunar site in a POM structure. At the same time, the coordination number of the imbedded metal cation should remain large in order to assure its interaction with the substrate. Due to their large coordination number, which is typically eight, Zr(IV) and Hf(IV) have been shown to be the most suitable choice for creating a Lewis acid active POM. Incorporation of Zr(IV) and Hf(IV) into POMs has led to catalysts that have been active in a wide range of different reactions such as the Mukaiyama aldol and Mannich-type additions (Boglio et al., 2007). The high Lewis acidity of Zr(IV)-POMs makes them also interesting as catalysts for the hydrolysis of the extremely stable phosphoester and peptide bonds, which are found in DNA and proteins, respectively. Our previous work has shown that Zr(IV) substituted Wells-Dawson [[Zr(α_2_-P_2_W_17_O_61_)_2_]^16−^, Zr1-WD2 (Vanhaecht et al., 2013), and [Zr_4_(α_2_-P_2_W_16_O_59_)_2_(μ_3_-O)_2_(OH)_2_(H_2_O)_4_]^14−^ POMs (Luong et al., 2016a), as well as Keggin POMs [(Et_2_NH_2_)_8_[{α-PW_11_O_39_Zr(μ-OH)(H_2_O)}_2_] and (Et_2_NH_2_)_10_[(α-PW_11_O_39_)_2_Zr] (Luong et al., 2014, 2015a,b, 2016b, 2017)], efficiently catalyze the cleavage of phosphoester bonds in nucleic acids as model substrates (Vanhaecht et al., 2013; Luong et al., 2014, 2015a,b, 2016a,b,c; Kandasamy et al., 2016). Also the selective cleavage of double-stranded DNA has been demonstrated (Luong et al., 2017). Interestingly, even though Zr(IV) and Hf(IV) have similar chemical properties and show a very similar coordination chemistry, the Hf(IV)-substituted Wells-Dawson POM showed a slightly higher reaction rate in the hydrolysis of phosphodiester bonds compared to the Zr(IV)-substituted Wells-Dawson POM (Zr1-WD2) (Vanhaecht et al., 2012). These POMs also exhibited hydrolyzing activity for a wide range of dipeptides (Absillis and Parac-Vogt, 2012; Ly et al., 2013a,b, 2016; Vanhaecht et al., 2013) oligopeptides (Absillis and Parac-Vogt, 2012; Vanhaecht et al., 2013; Ly et al., 2015b, 2016) and proteins (Stroobants et al., 2013, 2014a,b; Ly et al., 2015a; Sap et al., 2015a). Due to their negative charge POMs can specifically interact with positive regions of protein surfaces via electrostatic forces (Guangjin et al., 2007; Zhang et al., 2007, 2008; Zheng et al., 2009; Goovaerts et al., 2013), resulting in selective hydrolysis of peptide bonds located in the positive patches of proteins (Stroobants et al., 2014a). So far, several proteins such as hen egg white lysozyme (HEWL) (Sap et al., 2015b), human insulin β-chain (Sap et al., 2015a), human serum albumin (HSA) (Stroobants et al., 2014a,b), horse heart myoglobin (HHM) (Ly and Parac-Vogt, 2017), and cytochrome c (Cyt c) (Sap et al., 2016; Quanten et al., 2018) have been selectively hydrolyzed by Zr(IV)-substituted POMs (Stroobants et al., 2014a,b). Although these studies demonstrate the potential of Lewis acidic POMs to selectively hydrolyze peptide bonds in proteins, considering the very large variety of the possible structures and sizes of the proteins, further research is needed in order to understand the influence of these structural effects on the selectivity and efficiency of peptide bond hydrolysis. Specifically, the ability of highly negatively charged POMs to hydrolyze proteins with low pI and overall negative charge needs further investigation. Furthermore, except for one recent example (Vandebroek et al., 2018), nearly all the hydrolysis experiments have been performed with Zr-substituted POMs, while the protease activity of Hf-substituted POMs on proteins remains virtually unexplored. In this study, the reactivity of the Lewis acidic Hf(IV)-substituted Wells-Dawson POM, K_16_[Hf(α_2_-P_2_W_17_O_61_)_2_]·19H_2_O (Hf1-WD2, see Figure 1), was investigated toward the hydrolysis of a relatively large phosphorylated glycoprotein, ovalbumin (OVA). It is the most abundant protein in egg white, consisting of 385 amino acid residues and has a total molecular mass of 44.3 kDa. The secondary structural elements of OVA consist primarily of β-strands and α-helices. Moreover, ovalbumin (OVA) has a pI of 4.54 which means that at physiological pH, it has a net charge of −14, which might hinder the efficient binding to the Hf-POM catalyst. The interaction between OVA and Hf-POM has been investigated with a range of spectroscopic techniques in order to gain insight into the factors that influence the protein hydrolysis.

## Results and Discussion

### Reactivity of Hf1-WD2 in the Hydrolysis of OVA

**Figure 2:**
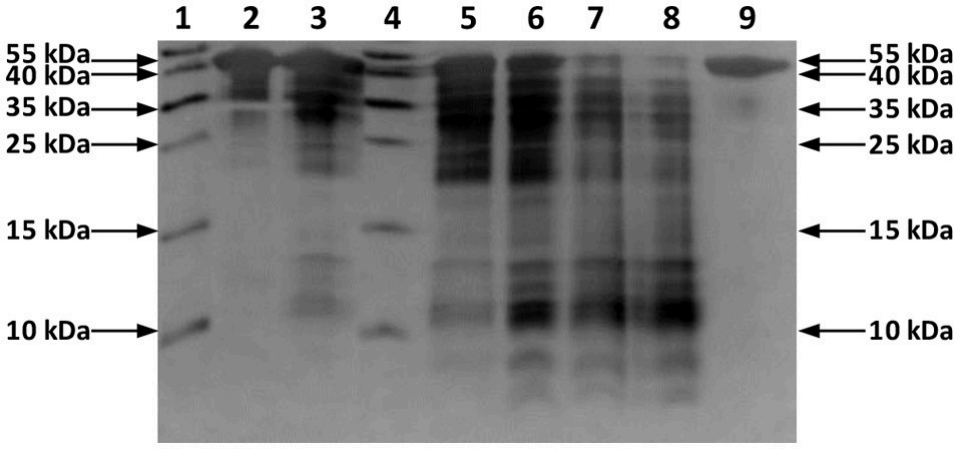
Silver-stained SDS-PAGE gel of OVA in the presence of the Hf1-WD2 POM. OVA (0.02 mM) was incubated with 100 equivalents of Hf1-WD2 at 60°C for up to 7 days in phosphate buffer (10.0 mM, pH 7.4). Lanes 1–9 from left to right: protein ladder, OVA and POM immediately after mixing, after 1 day, protein ladder, after 2, 4, 6, 7 days, and OVA only after 7 days.

In this study, a solution of OVA was incubated with Hf1-WD2 at 60°C in phosphate buffer (10.0 mM, pH 7.4). Aliquots of the reaction mixtures containing OVA (0.02 mM) and POM (2.0 mM) were taken at time intervals up to 7 days after mixing and were analyzed using sodium dodecyl sulfate polyacrylamide gel electrophoresis (SDS-PAGE) (see Figure 2). No precipitation was observed during the course of the reaction. New bands with a lower MW appeared on the SDS-PAGE gel after incubation for ~2 days at 60°C, indicating that hydrolysis of OVA had taken place in the presence of Hf1-WD2. To prove that Hf1-WD2 was indeed responsible for promoting hydrolysis of OVA, several control experiments were performed. In a first control experiment, OVA was incubated in the absence of POM up to 7 days at 60°C (Figure 2 right lane). Under these conditions, only the parent OVA protein was observed on the SDS-PAGE gel and there was no sign of hydrolysis. In a second control experiment, OVA was incubated for 7 days at 60°C with the α_2_-Wells-Dawson POM, which is similar to Hf1-WD2 except for the absence of the embedded Hf(IV) ion, but again no hydrolysis was observed. In a third control experiment, OVA was incubated with HfCl_2_O·8H_2_O salt for 7 days at 60°C. Under these conditions, only a minimal hydrolysis of OVA was observed, however also precipitation was observed in the reaction mixture (data not shown). All these tests clearly show that the Hf(IV) ion is a necessary component to induce hydrolysis, while the POM ligand serves as a stabilizing agent for Hf(IV) that prevents formation of insoluble gels in aqueous solution. To test whether Hf1-WD2 would be hydrolytically active toward OVA under both physiological temperature and pH, the temperature was lowered to 37°C, however very little hydrolysis was observed at pH 7.4 after 7 days. To evaluate the possible influence of the pH of the solution on the reactivity profile, the hydrolytic reaction was also followed in acetate buffer (10.0 mM, pH 4.4) and Tris-HCl buffer (10.0 mM, pH 9.0). Although one would expect that lowering the pH to pH 4.4 would result in improved interaction with Hf1-WD2 as the acidic environment increases the amount of positive charges on the OVA surface, interestingly only slight protein hydrolysis was observed. Similarly, no hydrolysis was revealed after incubation at 60°C and pH 9.0 for up to 6 days. The absence of hydrolysis might be due to the presence of precipitation which was observed under both these pH conditions and which probably resulted in the loss of Hf1-WD2 catalyst (see Figure S1). In addition, the presence of different buffers might also contribute to the observed changes in the reactivity, similarly to the recently reported inhibition and activation effect of buffers on the catalytic activity of Zr-POMs (Collins-Wildman et al., 2018).

### POM Speciation and Interaction Study

**Figure 3:**
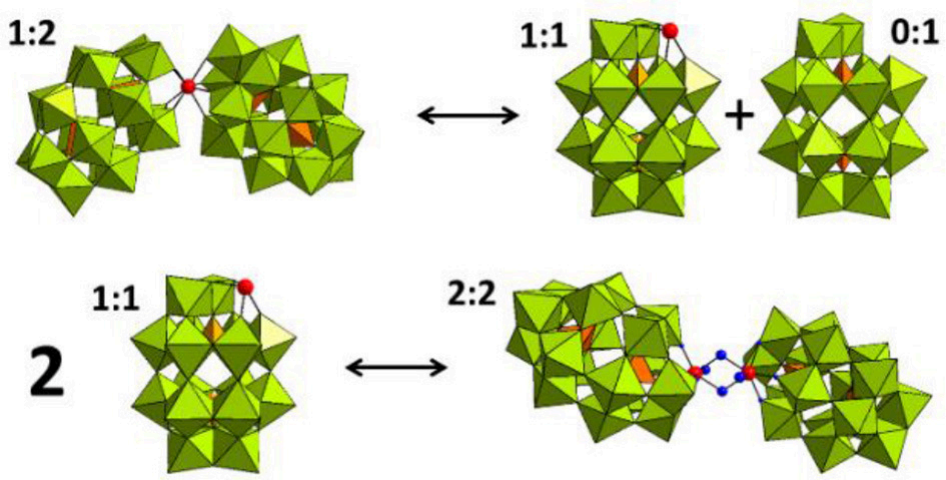
Equilibria between the 1:2, 1:1, and 2:2 species of the Hf(IV)-substituted Wells-Dawson POM. WO_6_ octahedrons are represented in olive green, PO_4_ tetrahedrons—in orange, and the Hf(IV) centers is represented as a red spheres.

**Figure 4:**
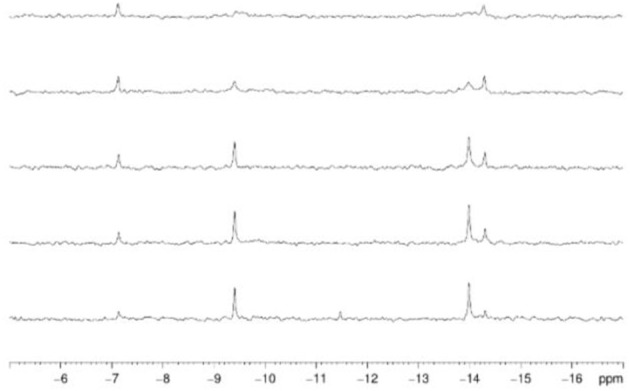
^31^P NMR spectra of Hf1-WD2 (2.0 mM) with increasing concentrations of ovalbumin in phosphate buffer (10.0 mM, pH 7.4, 10 % D_2_O) after mixing. From bottom to top: 0.0, 0.2 mM (10:1), 0.4 mM (5:1), 1.0 mM (2:1), 2.0 mM (1:1) of ovalbumin was added.

^31^P NMR spectroscopy was used to gain insight into the equilibria between the different POM catalyst species present in solution. In solution, Hf1-WD2 undergoes different equilibria which are highly influenced by factors such as concentration, pH, temperature and incubation time (see Figure 3). As can be deduced from Figure 4, Hf1-WD2 is presented as the 1–2 dimeric species in the absence of OVA, as indicated by the two peaks at −9.4 and −14.0 ppm (Kato et al., 2006). However, the additional appearance of two small signals at approximately −7.15 and −14.3 ppm indicate that the α_2_-Wells-Dawson species is also present in solution. The formation of the α_2_-Wells-Dawson POM appears to be induced by the phosphate buffer as these peaks are absent in D_2_O solutions that do not contain phosphate buffer (see Figure S2). In the presence of increasing concentrations of OVA, the signals corresponding to the α_2_-Wells-Dawson POM remain present (see Figure 4). However, the signals corresponding to the 1–2 dimeric species of Hf1-WD2 gradually broaden and completely disappear when the POM-protein molar ratio becomes 1:1. This fact is in a good relationship with the previously described mechanism of docking of the WD POM species on the surface of the proteins (Vandebroek et al., 2018). According to that knowledge, the broadening of the ^31^P NMR signals of Hf1-WD2 was observed because of the docking of the 1–2 dimeric species in the positive patch on the surface of OVA and the consecutive dynamic equilibria between the Hf1-WD2 dimer and the products of dissociation.

As the coordination sphere of Hf(IV) is fully saturated, it is unlikely that the dimeric 1:2 POM shown in the ^31^P NMR spectra is a catalytically active species. In contrast to the dimeric species, the 1–1 monomer does have free coordination sites available for binding to the protein substrate. It is therefore likely that a fast equilibrium takes place in solution in which the 1:2 dimeric species interconverts with the 1:1 monomeric species. In a recent study, we have shown that even though the dimeric Hf1-WD2 was mixed with HEWL, only the 1:1 monomeric species of the Hf(IV)-substituted Wells-Dawson POM was observed in a non-covalent complex with HEWL (Vandebroek et al., 2018). The single crystal X-ray structure of the non-covalent complex suggests that the monomeric POM species indeed can be formed in solutions containing a protein and the dimeric Hf1-WD2, and that it is the likely active catalytic species in protein hydrolysis experiments. ^31^P NMR measurements were also performed to investigate the stability and speciation of Hf1-WD2 in the presence of OVA throughout the hydrolytic reaction. As shown in Figure S3, Hf1-WD2 is stable in the presence of OVA during incubation for 7 days at 60°C and presents as the dimeric species.

### Interaction Between OVA and Hf1-WD2

Circular dichroism (CD) measurements show the presence of α-helical structure elements in OVA which are characterized by two minima at 208 nm and 222 nm. Titration experiments show that the α-helical structure is preserved upon addition of increasing concentrations of Hf1-WD2 (see Figure S4), suggesting that the interaction with the POM does not result in protein unfolding or denaturation.

**Figure 5:**
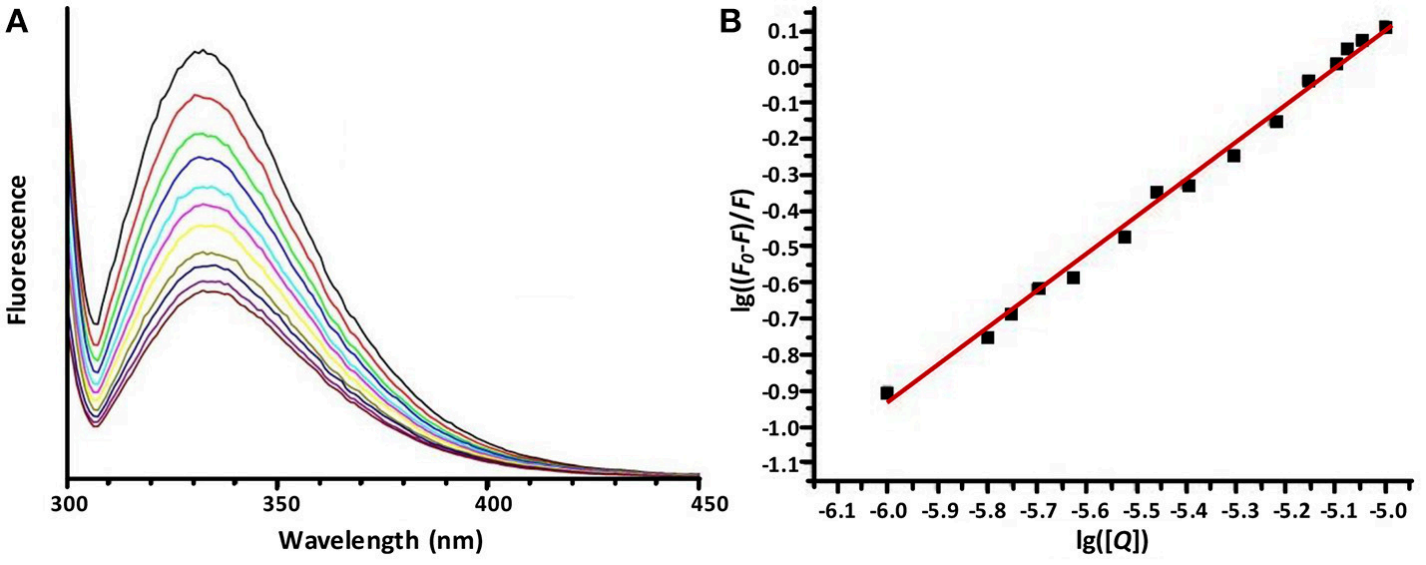
The emission spectra of OVA (10.0 μM) in sodium phosphate buffer (10 mM, pH 7.4) in the presence of increasing concentrations of Hf1-WD2. **(A)** demonstrates dependence of the emission fluorescence spectra (from top to bottom) from the concentration of Hf1-WD2, which was increased stepwise from 0 to 10 μM. All spectra were recorded from 300 to 420 nm while the sample was excited at 295 nm. The spectra are dominated by the Trp fluorescence with a maximum at around 337 nm. **(B)** displays the plot of the Stern-Volmer equation (with *R*^2^ = 0.976) that represents the quenched maximum fluorescence (lg((*F-F*_0_)/*F*)) in function of the logarithm of the concentration of the quencher (lg[*Q*]). From the plot, *K*_*q*_ and *n* were calculated to be 1.1 × 10^5^ M^−1^ and 0.85, respectively.

The interaction between OVA and Hf1-WD2 was further studied using tryptophan (Trp) fluorescence quenching. OVA contains three tryptophan (Trp) and ten tyrosine (Tyr) residues, however despite the presence of ten Tyr residues, its emission spectrum is dominated by Trp fluorescence (see Figure 5), since Trp absorbs at higher wavelengths where Tyr no longer absorbs (above 290 nm). Moreover, any energy absorbed by Tyr residues is often efficiently transferred to the Trp residues in the same protein (Lakowicz, 2007). Therefore, using excitation light at 295 nm avoids any detectable emission of the Tyr residues. Trp fluorescence is widely used as a tool to monitor changes in the local protein environment and to study protein-POM interactions (Goovaerts et al., 2015a,b; Ly and Parac-Vogt, 2017).

As Figure 5 shows, the Trp fluorescence of OVA is quenched by the addition of Hf1-WD2, indicating that binding took place. This quenching can be fitted to the derived Stern-Volmer equation (**1**) (Hungerford et al., 2010):

(1) $$\begin{array}{r} lg\left(\frac{\left(F_{0}-F\right)}{F}\right)=lgK_{q}+n\cdot lg\left(\left[Q\right]\right) \end{array}$$

Herein *F*_0_ represents the unquenched fluorescence, *F* the quenched fluorescence, [*Q*] the concentration of the quencher, *K*_*q*_ the quenching constant and *n* the number of bound quenching molecules to the protein. According to equation (**1**), the calculated values of *K*_*q*_(M^−1^) and *n* are (1.1±0.3) × 10^5^ and 0.85 ± 0.04 (*R*2 = 0.976), respectively, for the interaction between OVA and Hf1-WD2 (see Table 1).

**Table 1:** Calculated values of the quenching constants (*K*_*q*_), their corresponding number of bound molecules (*n*), the percentage of hydrolyzed OVA and HSA (Stroobants et al., 2014a) after 48 h incubation at pH 7.4 and 60°C for different POM-protein complexes.

**Protein**	**POM**	***K_***q***_* (M^**−1**^)**	***n***	**Hydrolysis (%) after 48 h**
OVA	Hf1-WD2	1.1 × 10^5^	0.85	~50 (This study)
HSA	Zr1-WD2	5.1 × 10^5^	1.52	~75 (Stroobants et al., 2014a)

**Figure 6:**
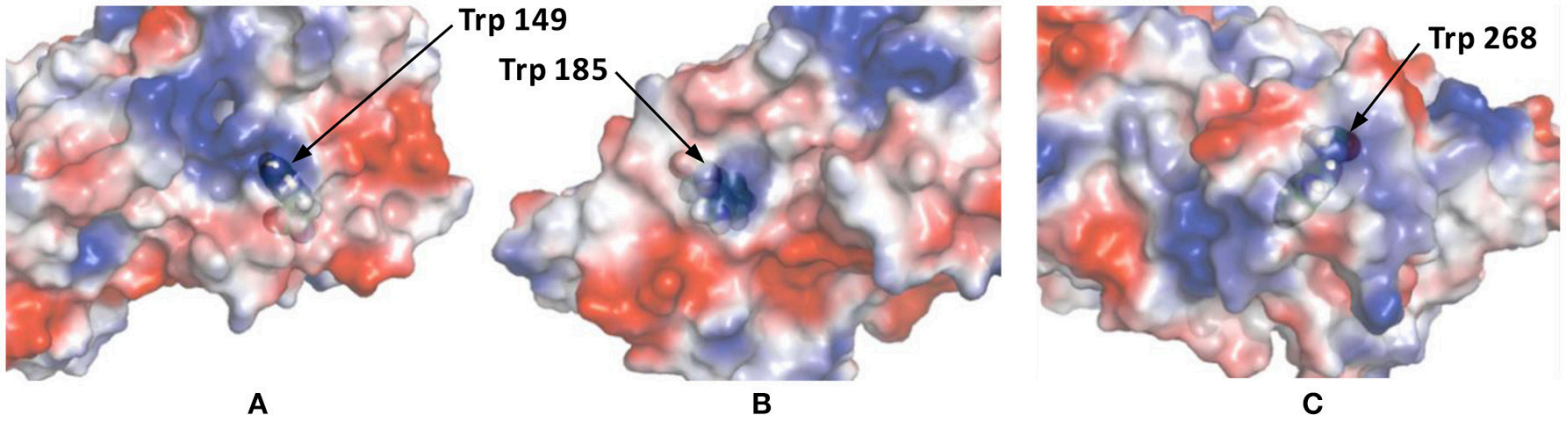
The surface charge of OVA as rendered by PyMol's Vacuum Electrostatics. Red, white and blue stand for negative, neutral and positive surface charges, respectively. **(A)** displays Trp residue 149 and is solvent accessible through a positive surface patch. **(B)** shows Trp residue 185, which is not solvent accessible, but is still in close proximity of a positive patch. The final Trp residue, 268, is shown in **(C)**. This solvent accessible Trp residue is located in a small, positive surface patch.

This *K*_*q*_ value is in the same range of magnitude as the *K*_*q*_ which was found for the binding between Zr1-WD2 and HSA [*K*_*q*_(M^−1^) = 5.1 × 10^5^] (Goovaerts et al., 2015b). The slightly stronger interaction observed in the case of HSA might be due to the larger size of HSA (585 amino acids and 66.5 kDa compared to 385 amino acids and 44.3 kDa for OVA), which is noticeable by the smaller number of POMs that OVA can accommodate (0.85 for OVA compared to 1.5 for HSA). The second factor is the higher pI of HSA than OVA (5.2 and 4.5, respectively) leading to a less negative total surface charge of HSA (about-12) compared to OVA (about-14) at the experimental pH (7.4). Nevertheless, the positively charged patch on the OVA surface presents even at pH = 7.4 and defines the selectivity of docking of the POM species on the surface of the protein. The size of the positive patch on the surface of OVA is 1.3 × 1.0 nm, that correlates quite well with the size of the monomeric POM species (1.2 × 1.0 nm) and significantly smaller than the size of the dimeric species (about 3.0 × 1.4 nm). The single Trp residue in HSA is located inside a highly positive cleft which can nicely accommodate a Zr1-WD2, which is not the case for Trp residues found in OVA. While two of the three Trp residues in OVA are accessible for a solvent and are located near a positively charged surface area (see Figure 6), the third Trp residue (Trp185) is not solvent accessible, but it is in close proximity to the surface of the protein. Considering that the peak position and profile do not change as more POM is added, it is reasonable to assume that all Trp residues are affected by the binding of the POM (Lakowicz, 2007). One of the likely binding sites is close to Trp149 as this residue is located in an easily accessible and wide surface area. While binding close to Trp268 cannot be excluded, interaction around this residue is less likely as this residue is surrounded by negatively charged regions.

### Selectivity of Hydrolysis

To identify the exact cleavage sites in OVA induced by Hf1-WD2, the protein fragments from the SDS-PAGE gel were first transferred to a polyvinylidene fluoride (PVDF) membrane which was then Coomassie-stained. Thereafter, Edman degradation was performed on the Coomassie-stained protein fragments which demonstrated that Hf1-WD2 POM selectively hydrolyzed OVA at Phe13-Asp14, Arg85-Asp86, Asn95-Asp96, Ala139-Asp140, Ser148-Trp149, Ala361-Asp362, Asp362-His363, and Pro364-Phe365 bonds (see Figures S5, S6, Table S1).

All hydrolyzed peptide bonds in OVA are located in the positively charged surface regions of the protein to which the negatively charged Wells-Dawson POM can easily dock prior to hydrolysis. Moreover, all observed cleavage sites always contained Asp, either as part of the hydrolyzed peptide bond (Asp-X or X-Asp, see Figures S5, S6) or in the close proximity to the hydrolyzed peptide bond. Interestingly, there are nine X-Asp bonds present in OVA which are not hydrolyzed by Hf1-WD2, which can most likely be attributed to these sites being sterically inaccessible.

The affinity of Hf1-WD2 to hydrolyze peptide bonds in the vicinity of Asp residues is consistent with the previous studies involving Zr-POM catalysts. For example, it was shown that three out of the four hydrolyzed peptide bonds in HSA were at X-Asp or X-Glu (Stroobants et al., 2014a). Similarly, HHM was exclusively hydrolyzed at Asp-X peptide bonds by different Zr(IV)-POMs (Ly et al., 2015a). Furthermore, two out of three hydrolysis sites in Cyt c hydrolyzed by Zr(IV)-substituted Keggin POMs were also cleaved at Asp-X peptide bonds (Sap et al., 2016), and a recent study has shown that Hf1-WD2 hydrolyzed HEWL at nine sites, all of which were Asp-X or X-Asp (Vandebroek et al., 2018). In all cases, the hydrolyzed peptide bonds were situated close to a positively charged patch of the protein to which the negatively charged POM can dock. The selectivity of POMs toward peptide bonds in the proximity of an Asp residue is not yet fully understood and is currently being investigated with the help of theoretical methods. It is however plausible that the carboxylate group found in the side chain of Asp can assist hydrolysis either via direct intramolecular nucleophilic attack on the carbonyl carbon, or via accepting protons from water molecules which then act as effective nucleophiles. In either cases the interaction between the Lewis acid metal ion [Zr(IV), Hf(IV)] and the carbonyl oxygen of the peptide bond is essential as it polarizes carbonyl bonds and makes carbon atoms more susceptible for nucleophilic attack (Ly et al., 2015c; Mihaylov et al., 2016).

## Conclusions

In this study, the Hf(IV)-substituted Wells-Dawson POM, K_16_[Hf(α_2_-P_2_W_17_O_61_)_2_] (Hf1-WD2) was shown to act as an efficient and site-selective artificial protease for the hydrolysis of OVA. In accordance to previously studied proteins, the hydrolysis preferentially occurs in the vicinity of Asp residues located in positively charged patches of proteins. A combination of POM-protein electrostatic interactions and Asp side chain assisted nucleophilic attack on the carbon atom of the peptide bond are likely at the origin of the observed selectivity. The Hf(IV) and Zr(IV) substituted Wells-Dawson POMs seem to show similar selectivity in hydrolysis experiments, which can be attributed to their similar structures and a similar Lewis acidic behavior of these two metal ions. Interestingly, the highly negatively charged Hf1-WD2 was able to hydrolyze OVA protein which has a relatively low pI of 4.54 and an overall negative charge of −14 under physiological pH. This suggests that for the hydrolysis to occur the overall charge of the protein is not the limiting factor as long as the protein contains the positively charged patches which are accessible for interaction with the POM catalyst.

## Experimental

### Materials

Albumin from chicken egg white lyophilized powder (ovalbumin, OVA), deuteriumoxide (D_2_O), N,N,N′,N′-tetramethylethylenediamine (TEMED), and disodium phosphate (Na_2_HPO_4_) were bought from Sigma-Aldrich. Hafnium oxychloride octahydrate (HfOCl_2_·8H_2_O) and diethyl ether (Et_2_O) were purchased from ChemLab. Aqueous hydrochloric acid (HCl, 37%), acetic acid (CH_3_COOH), sodium acetate (CH_3_COONa), and potassium hydrogen carbonate (NaHCO_3_) were obtained from Acros Organics. Methanol and monosodiumphosphate (NaH_2_PO_4_) were purchased from VWR. Ethanol, aqueous orthophosphoric acid (H_3_PO_4_, 85%), and protein ladders were acquired from Thermo Fisher Scientific. Potassium chloride (KCl), tris(hydroxymethyl)aminomethane (TRIS), and acrylamide:bisacrylamide (29:1) solution (30%) were procured from AppliChem. All compounds were purchased in the highest available purity and were used without further purification. α-/β-K_6_P_2_W_18_O_62_·14/19H_2_O (Contant et al., 1990), α_2_-K_10_P_2_W_17_O_61_·20H_2_O (Contant et al., 1990), and K_16_[Hf(α_2_-P_2_W_17_O_61_)_2_]·19H_2_O (Kato et al., 2006) were synthesized according to the reported literature procedures.

### Methods

#### Hydrolysis Study

Solutions containing OVA (0.02 mM) and K_16_[Hf(α_2_-P_2_W_17_O_61_)_2_]·19H_2_O (2.0 mM) were prepared in phosphate buffer (10 mM, pH 7.4), acetate buffer (10 mM, pH 4.4), or Tris-HCl buffer (10 mM, pH 9.0) Samples were incubated at 60 or 37°C and aliquots were taken at different time increments and analyzed by SDS-PAGE.

#### Electrophoresis

SDS-PAGE was performed on a 16% (wt./vol.) polyacrylamide resolving gel (Tris-HCl buffer, 1.5 M, pH 8.8) and a 4% (wt./vol.) polyacrylamide stacking gel (Tris-HCl, 0.5 M, pH 6.8). Each sample (15 μL) was mixed with sample buffer (5 μL) and heated to 95°C for 5 min. A volume of 10 μL of the resulting solution was loaded onto the gel. A PageRuler prestained protein ladder (10–170 kDa) was used as a molecular mass standard. An OmniPAGE electrophoretic cell was used with an EV243 power supply (both produced by Consort). Two SDS-PAGE gels were run at the same time in a Tris-Glycine-SDS running buffer with the maximum voltage set to 200 V, a constant current set to 70 mA, and the maximum power set to 50 W. The total running time was ~1.5 h. SDS-PAGE gels were visualized with silver staining or coomassie staining and an image of each gel was taken using a GelDoc EZ Imager (BioRad).

#### Edman Degradation

SDS-PAGE-separated proteins were blotted onto a PVDF membrane (using a BioRad Trans-Blot Turbo RTA Transfer Kit) and stained with Coomassie blue. The bands were cut from the stained membrane, destained in methanol, rinsed with ultrapure water, and then subjected to automated NH_2_-terminal amino acid sequence analysis (Procise 491 cLC protein sequencer, Applied Biosystems, Foster City, CA) based on the Edman degradation reaction (Loos et al., 2009).

#### ^31^P NMR Spectroscopy

All ^31^P NMR spectra were recorded on a Bruker Avance 400 (161.98 MHz) spectrometer. As an external standard, 25% H_3_PO_4_ in D_2_O in a sealed capillary was used. Upon mixing: Solutions containing K_16_[Hf(α_2_-P_2_W_17_O_61_)_2_]·19H_2_O (2.0 mM) and increasing concentrations of OVA (0, 0.2, 0.4, 1.0, and 2.0 mM) were prepared in phosphate buffer (10.0 mM, pH 7.4, 10% D_2_O) and measured directly after mixing. During the reaction: A solution containing K_16_[Hf(α_2_-P_2_W_17_O_61_)_2_]·19H_2_O (2.0 mM) and OVA (0.4 mM) was prepared in phosphate buffer (10.0 mM, pH 7.4, 10% D_2_O) and was measured after mixing and after incubation for 7 d at 60°C.

#### Circular Dichroism Spectroscopy

Solutions containing OVA (5.0 μM) and K_16_[Hf(α_2_-P_2_W_17_O_61_)_2_]·19H_2_O (Hf1-WD2) (0 to 25 μM) were prepared in phosphate buffer (10.0 mM, pH 7.4). CD measurements were performed using a JASCO-1500 directly after preparation of the samples. The samples were analyzed in quartz cells with a path length of 1.0 mm. Scans were recorded in the far-UV wavelength region (185–260 nm) where peptide bond absorption takes place. All CD spectra were corrected for the background effect by subtracting the spectrum of the respective buffer solution from the spectrum of the protein.

#### Fluorescence Spectroscopy

Steady state fluorescence was measured on an Edinburgh Instruments FS900 spectrofluorimeter, using quartz cuvettes with a 10.0 mm optical path length. Tryptophan fluorescence spectra of buffered 10.0 μM protein solutions (pH 7.4, 10.0 mM sodium phosphate buffer), were recorded at ambient temperature ranging from 300 to 450 nm, with a maximum at ~330 nm. The samples were excited at 295 nm to avoid excitation of the tyrosine residues. Each emission spectrum was recorded in threefold and averaged to take any random error into account. The concentration of the POMs was increased from 0 to 10.0 μM in 1.0 μM steps.

## Author Contributions

All experimental work was performed by AS and TQ under guidance of TP-V and PP. Edman degradation was performed by PP. Data analysis was performed by AA, AS, and TQ with valuable contributions and corrections of TP-V and PP. The manuscript was written by AA with valuable contributions and corrections from TP-V and PP.

### Conflict of Interest Statement

The authors declare that the research was conducted in the absence of any commercial or financial relationships that could be construed as a potential conflict of interest.
