# Supplementary material for: Selective Hydrolysis of Ovalbumin Promoted by Hf(IV)-Substituted Wells-Dawson-Type Polyoxometalate
Source: Front Chem. 2018 Dec 13;6:614. doi: 10.3389/fchem.2018.00614 (PMC6305993; doi:10.3389/fchem.2018.00614)
Supplement: Supplementary file 1 [file Presentation_1.pdf]

# *Supplementary Material*

# Selective Hydrolysis of Ovalbumin Promoted by Hf-substituted Wells-Dawson-Type Polyoxometalate

**Alexander V. Anyushin, Annelies Sap, Thomas Quanten, Paul Proost, and Tatjana N. Parac-Vogt***

*** Correspondence:** Corresponding Author: tatjana.vogt@kuleuven.be

## 1 Supplementary Figures

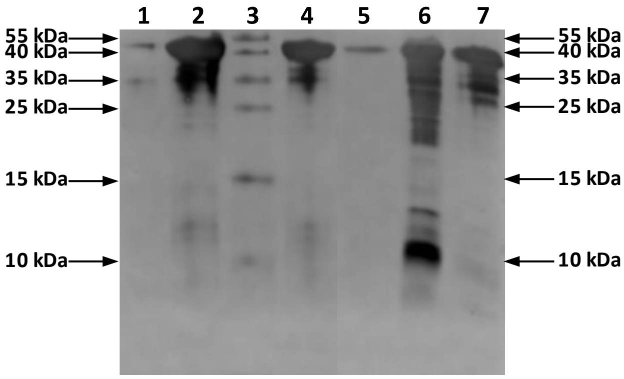


**Figure *S1*.** Silver-stained SDS-PAGE gel of OVA in the presence of Hf1-WD2.
OVA (0.02 mM) was incubated with 100 equivalents of Hf1-WD2 for 2 days at 60 °C in phosphate buffer (10.0 mM, pH 7.4), acetate buffer (10.0 mM, pH 4.4) or Tris-Cl buffer (10.0 mM, pH 9.0). Lanes 1-10 from left to right: (1) OVA only at pH 4.4, (2) OVA only at pH 7.4, (3) protein ladder, (4) OVA only at pH 9.0, (5) OVA + Hf1-WD2 at pH .4.4, (6) OVA + Hf1-WD2 at pH 7.4, (7) OVA + Hf-1WD2 at pH 9.0.

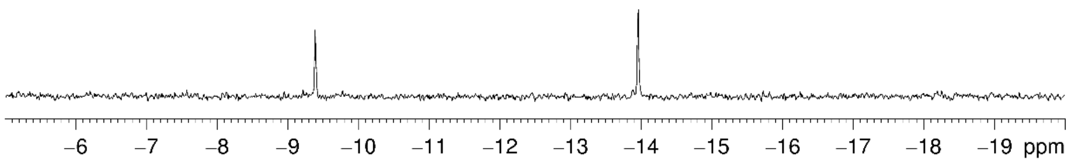


**Figure *S2*.** $^{31}$P NMR spectrum of Hf1-WD2 POM in 100 % $D_2O$ at RT.

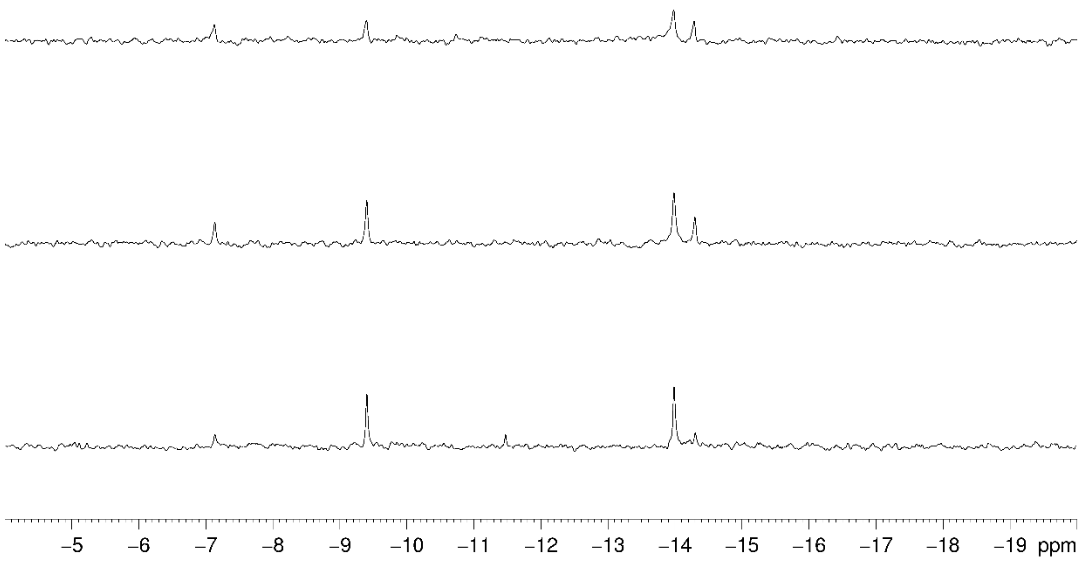


**Figure *S3*.** $^{31}$P NMR spectra of Hf1-WD2 in phosphate buffer.
The $^{31}$P NMR spectra of Hf1-WD2 (2.0 mM) is shown in phosphate buffer (10.0 mM, pH 7.4, 10 % $D_2O$) without OVA at RT (bottom), with 0.4 mM OVA at RT (middle), and with 0.4 mM OVA after 7 days at 60 °C (top).

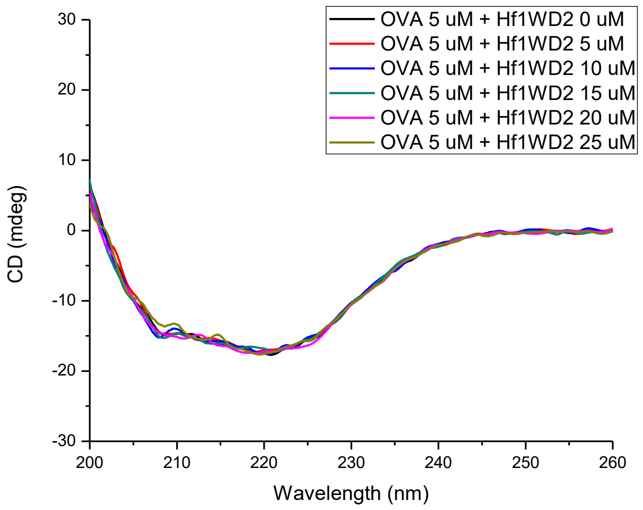


**Figure *S4*.** CD plot of OVA (5.0 uM) with increasing concentrations of Hf1-WD2 in phosphate buffer (10.0 mM, pH 7.4) after mixing.

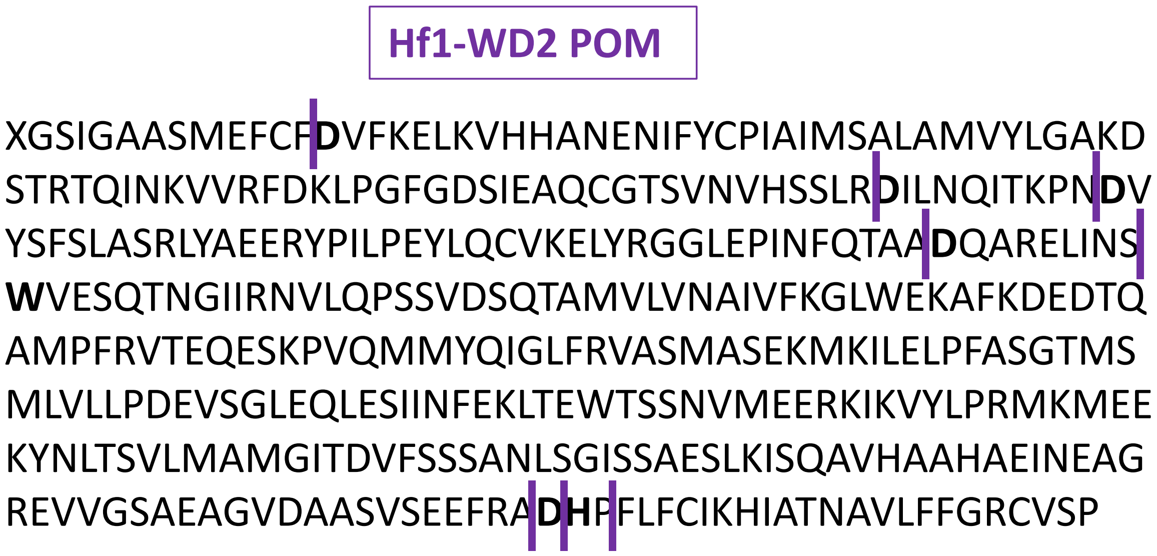


**Figure *S5*.** Primary amino acid sequence of OVA with the hydrolysis sites induced by Hf1-WD2 POM indicated in purple.

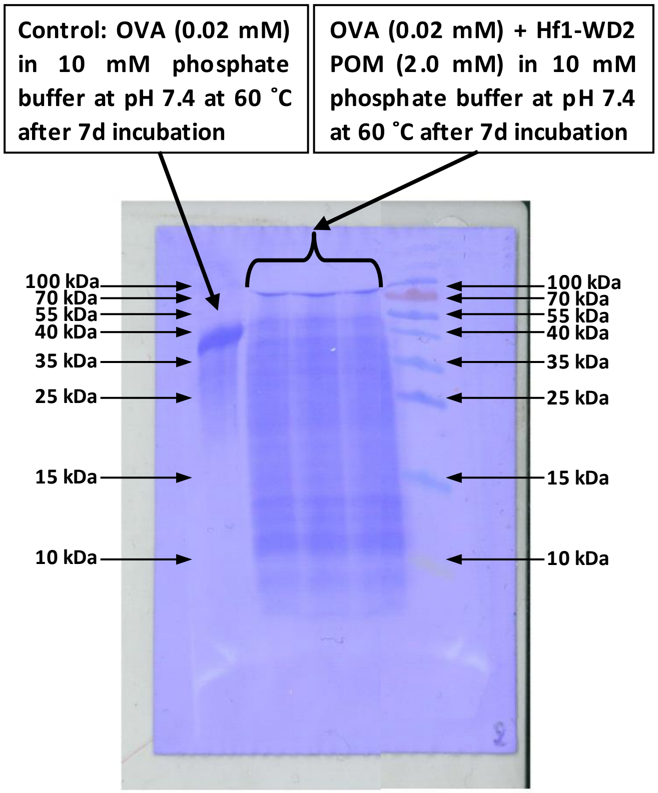


**Figure *S6*.** Coomassie stained blot of OVA hydrolysis by Zr1-WD2 POM or Hf1-WD2 POM.
An SDS-PAGE gel of OVA (0.02 mM) hydrolyzed by Zr1-WD2 POM or Hf1-WD2 POM (2.0 mM) after incubation in phosphate buffer (10.0 mM, pH 7.4) for 7d at 60 °C was blotted on a PVDF membrane and Coomassie stained.

## 2 Supplementary Tables

**Table S*1*.** Overview of N-terminal amino acid sequences determined by Edman Degradation and the corresponding peptide bonds hydrolyzed by Hf1-WD2 POM.

| **Sequence determined by Edman degradation** | **Hydrolyzed peptide bond** |
|---|---|
| DHPFLFXIXH | Ala361-Asp362 |
| HPFLFXI | Asp362-His363 |
| FLFXIXXIAT | Pro364-Phe365 |
| DVFXELXV | Phe13-Asp14 |
| XVESQTN | Ser148-Trp149 |
| DQAXELINS | Ala139-Asp140 |
| DVYSFSLAS | Asn95-Asp96 |
| DILNQ | Arg85-Asp86 |
